# Mechanism of career resilience formation during the role transition of medical interns: a grounded theory study

**DOI:** 10.3389/fmed.2025.1671520

**Published:** 2025-11-17

**Authors:** Jinyu Zhou, Lifu Jin, Yamin Hu

**Affiliations:** 1 School of Management, Jiangsu University, Zhenjiang, Jiangsu, China; 2 Student Affairs Section, School of Medical Technology, Xi’an Medical University, Xi’an, Shaanxi, China

**Keywords:** interns, career resilience, role transition, proactive career adaptation, grounded theory

## Abstract

**Background:**

The complexity, difficulty and uncertainty inherent in the medical field pose significant challenges to physicians’ ability to adapt. For interns, career resilience plays a crucial role in the transition from academia to clinical practice. This study aimed to explore the mechanism of career resilience formation during the role-transition process of medical interns.

**Methods:**

We conducted in-depth semi-structured interviews with 16 medical interns from a regional medical university in Northwest China, recruited via purposive and snowball sampling through professional networks. Data were collected between August and September 2024; interviews lasted 35–50 min (online/offline), were audio-recorded, transcribed verbatim, and analyzed using Straussian grounded theory (open–axial–selective coding with constant comparison), guided by the Critical Incident Technique and the STAR framework. The research team performed the coding, and the process was validated through regular peer-debriefing sessions with two independent researchers. Analysis was facilitated using NVivo software (version 12) until theoretical saturation was reached.

**Results:**

A Challenge-Resource-Adaptation (CRA) model was constructed to explain how medical interns develop career resilience in the face of practical challenges and role transitions during the internship. Three pillars of career resilience were identified: (1) career development challenges (knowledge updating, identity establishment, career planning); (2) facilitative resources (guidance and feedback, resources and opportunities, emotional support); and (3) proactive career adaptation (compliance/pressure management and innovative breakthrough). The CRA model depicts a recursive loop whereby each innovative breakthrough elevates demands for further knowledge updating. Illustratively, weekly mentor-guided guideline reviews and skills-lab rehearsal closed knowledge-practice gaps, enabling independent ward rounds; exposure to advanced procedures helped clarify specialty choices.

**Conclusion:**

Career development challenges are the trigger factor, facilitative resources are the favorable conditions, and career adaptation behavior is the external manifestation of resilience. Overall, career resilience during internship emerges as a self-reinforcing process in which targeted resources convert concrete challenges into adaptive behaviors. The CRA model identifies mentor feedback, advanced clinical opportunities, and emotional support as key leverage points for strengthening medical curricula and workplace design.

## 1 Introduction

Medical internships place trainees in complex, high-stakes, and uncertain clinical settings where role expectations shift rapidly (1, 2). In this context, career resilience is central to interns’ successful role transition from student to clinician (3, 4).

During their internship, medical interns enter an inherently complex, high-pressure, and uncertain clinical environment (2). Career resilience, which in this study is defined as interns’ dynamic capacity to recover from professional setbacks (5) and to proactively adapt their behaviors to sustain motivation during the role-transition period (6), has emerged as a vital resource for fostering long-term professional growth (3).

Moreover, in China, medical students face persistent psychological stress due to long academic cycles, intensive clinical demands, and systemic factors such as promotion pressures, low incomes, and strained doctor-patient relationships (7). Compared with students of other majors, medical students have significantly higher stress in clinical practice, mainly due to academic load, career uncertainty, and interpersonal conflict (8). Grush et al. pointed out that secondary traumatic stress was significantly higher in medical students during clinical rotation, and the internship period constituted a high incidence window for psychological stress (9). At the same time, qualitative research further revealed that the “baptism of responsibility” in the process of medical practice often brings sudden mood swings and adjustment difficulties (10). As for the mechanism of stress, a large number of studies have pointed out that uncertainty is the key factor in stimulating avoidance behavior and cognitive confusion (11). Long-term exposure to high-uncertainty fields will cause chronic depletion of the nervous system and may induce disease (12). These pressures raise a practical question for medical education about how interns build career resilience during internship rather than merely cope with stress. Prior research has largely focused on psychological resilience, whereas the formation mechanism of career resilience in clinical practice remains insufficiently explored (8, 13, 14).

In this study, we focus specifically on career resilience during the student-to-intern transition and examine how concrete incidents and supports translate into adaptive strategies and accumulating competence. Developing career resilience during medical students’ clinical internships is critical to effectively addressing complex medical environments and career transition challenges (3). In this study, career resilience denotes clinical interns’ capacity to recover from setbacks, maintain motivation, and continue professional growth in demanding healthcare settings. Although studies have explored career readiness and expectations of medical graduates, further research is needed to understand the unknown difficulties encountered by clinical interns in their transition from “medical student” to “intern” and their development of career resilience (1).

This study aims to address this gap by developing an empirically grounded explanation of how career resilience emerges and evolves during medical internship. Accordingly, this study employs a grounded theory approach to explore how career resilience develops during role transition. In view of this, this study conducted in-depth interviews with 16 medical students in clinical practice with the following research questions:

(1)    What are the core dimensions and specific elements of medical interns’ career resilience during clinical practice?
(2)    How are these dimensions interrelated to form a mechanism of resilience formation during role transition?
(3)    What roles do challenges, resources, and adaptive behaviors, respectively, play in cultivating interns’ career resilience, thereby indicating targets for educational intervention?

## 2 Literature review

### 2.1 Resilience framework in international medical education research

The concept of resilience originated in the field of systems safety and spread rapidly in international research to other disciplines such as complex science, ecology, organization management, and psychology, especially in psychology, from which “psychological resilience” derived similar concepts such as resilience and stress tolerance (15). However, in the field of medical education, few studies have expanded the concept of resilience. In these discussions, it was clearly pointed out that “academic resilience” is an important concept that affects the academic growth of medical students, especially when it comes to factors that hinder or promote academic development. For example, in the context of medical students, the ability to overcome intellectual or technical skill limitations is a critical life skill (16). When discussing the barrier factors and facilitating factors affecting medical students’ academic development, the concept of “academic resilience” was clearly put forward, and it was considered that, besides cognitive skills and technical skills, resilience is an extremely important life skill for medical students to cope with academic difficulties. Academic resilience not only supports the academic growth of medical students but also facilitates their transition into clinical practice, suggesting an extension of the construct from “academic resilience in medical students” to “career resilience in early-career physicians (17).

Researchers usually infer resilience by assessing risks or disadvantages and then determining whether subjects overcome setbacks caused by disadvantages and are able to adapt positively (15). These findings suggest that objective risk factors and subjectively acquired positive adjustment constitute two essential components in the formation of resilience. Moreover, Sandler (18) emphasizes that resilience resources operating at individual, micro-, and macro-levels buffer the impact of adversity by satisfying human needs and offsetting its negative consequences (18). Protective mechanisms play an important role in adversity by reducing the effects of risk and enhancing self-esteem and self-efficacy (19). All of the above studies demonstrate the supportive role of protective factors in shaping resilience. The above research indicates that adversity, external resources and support, and individual adaptation are the three indispensable pillars of a general resilience-formation framework—providing the theoretical basis for examining how medical students develop career resilience during clinical practice.

### 2.2 Career resilience in the professional development of doctors

Career resilience in the face of adversity has been widely recognized as a core physician competency (20). It is viewed both as a personal trait that fosters professional development and as an ideal quality sought by healthcare organizations. From a competency perspective, professionals involved in major national medical programs identified diligence, persistence, and hard work as the most important qualities for doctors (21). They further noted that professional knowledge, communication and collaboration skills, and humanities literacy also underpin physicians’ career resilience. From a developmental perspective, doctors progress through six stages from enlightenment to maturity (22). Although external factors—such as family, education, and organizational context—vary in influence across these stages, personal traits remain a constant driver of career resilience. In particular, “hardship,” “adaptation,” “effort,” and “endurance” are frequently cited by good doctors as factors that are inextricably linked to their career resilience (15). From the standpoint of societal expectations, a willingness to embrace hard work and patience is are indispensable professional characteristic of excellent doctors, and these qualities also externally manifest physicians’ career resilience (23). In addition, studies have pointed out that educators should cultivate resource-rich, resilient, and responsible 3R doctors through medical education reform, help medical students adapt to complex medical environments, and emphasize the important role of career resilience in cultivating future-oriented medical talents (3). A study of multinational medical student samples found that mental interventions such as mindfulness training and cognitive behavioral therapy can improve career resilience, and relieve stress and anxiety in the short term, but the long-term improvement effect on depression and quality of life is uncertain (24). Structured career resilience training programs developed in a multidisciplinary setting have been shown to significantly improve medical students’ self-rated resilience, motivation, and social support, as well as their ability to apply protective factors in clinical settings (25). Intervention studies have tested that systematic peer counseling can effectively enhance the career resilience and self-efficacy of medical students, improve their ability to adapt to high-pressure clinical environments, and maintain mental health (4, 26). Recent research, represented by SAR models, has shown that systematic integration of career resilience training programs into both curriculum and learning environments can significantly improve medical students’ self-management, peer collaboration, and mental health (27).

### 2.3 Theoretical foundations and gaps in general resilience models

Classic theories conceptualize resilience chiefly as re-integration to equilibrium. Richardson’s Resiliency Model posits that adversity disrupts biopsychospiritual homeostasis and individuals subsequently “reinstate or elevate” their prior functioning (28). One study underscored the interplay between risk exposure and protective assets, yet still conceived positive adaptation as a return to baseline (29). Systematic critiques contend that such “bounce-back” models rarely theorize recursive growth following repeated successes. In medical-education contexts that feature rapid technology turnover and escalating clinical complexity, a mere “restoration” lens is insufficient (30). What remains under-theorized is how each successful adaptation generates higher-order challenges, prompting new learning cycles—a pattern repeatedly observed in internship trajectories. To address this theoretical gap, the present study advances a recursive spiral model in which each Innovative Breakthrough triggers Knowledge Updating and subsequently elicits higher-order challenges, thereby portraying career resilience as an upward, self-reinforcing trajectory rather than an end-state recovery.

### 2.4 Limitations of existing research

Although the positive role of career resilience in doctors’ career development has been recognized, the introduction of resilience theory into medical education and the exploration of medical students’ career resilience in clinical practice remain limited. There are a few studies in the field of international medical education that attempt to correlate variables related to resilience in the context of medical learning, but these studies only focus on verifying the correlation between resilience and medical emotion (31), mental health (32), and academic achievement (33), without considering the specific mechanism of resilience formation. Therefore, this study introduces the concept of career resilience into medical education, follows a bottom-up inductive logic typical of exploratory research, and adopts a qualitative design to explore the formation mechanism of medical students’ career resilience in clinical practice.

In summary, prior research has demonstrated the significance of resilience in medical education but has seldom examined how career resilience develops through the lived experiences of medical interns. Existing studies mostly verify correlations among related variables, overlooking the underlying process by which resilience takes shape in clinical contexts. Addressing this limitation requires an inductive, experience-based exploration. Therefore, the present study applies a grounded-theory approach to construct an empirically grounded explanation of the formation of career resilience during the internship stage, which provides the foundation for the following research design.

### 2.5 Research design

The concept of career resilience in clinical interns is still without a clear, shared definition. Consequently, the specific components and formation mechanisms of career resilience remain under-explored in medical-education research. Because career resilience takes shape through success and failure experiences, we employed a qualitative design grounded in key events. This approach allowed us to capture the rich workplace settings and the interns’ lived experiences in vivid detail. Before the interview, participants were provided with a detailed explanation of the research objectives and interview content. They were then asked to sign a “Research Participation Consent Form,” and the interviews commenced only after their consent was obtained. Clinical internships are a stress-intensive phase of medical education characterized by placing significant emotional and physical demands on students’ adaptability and affecting their quality of professional life (4). As students transition from theoretical learning to real clinical settings, they encounter workplace realities that are critical for career resilience under stress and the development of professional identity (34).

#### 2.5.1 Ethics statement

The study protocol was reviewed and approved by the Xi’an Medical University Medical Ethics Review Committee (Approval No. XYLS2024221). All participants provided written informed consent before participation.

### 2.6 Sample characteristics

In terms of sample selection criteria, referring to the requirements of scholars Stassen and Westerman (35) on the working hours of doctors in the early stage of their careers, and combining with the actual situation of the internship period in medical colleges, four conditions were set: ➀ They had completed the study of theoretical courses in schools; This is consistent with previous research emphasizing that the shift from classroom to clinical settings marks a major shift in role responsibility and stress exposure (36). ➁ They were currently on probation and internship for 1 year. This phase has proven to be critical for developing resilience and dealing with workplace demands (4). ➂ They had participated in the work in medical places. Research has demonstrated that true clinical responsibility under supervision is key to adaptive learning and stress management (37). ➃ They were in the undergraduate or postgraduate study stage. Internship stress was recorded at both academic levels during undergraduate and graduate study (38). According to this standard, participants were recruited primarily through the researchers’ own professional networks, including colleagues and supervisors at medical institutions. The recruitment process was further expanded using snowball sampling, where initial participants recommended other eligible medical students. To ensure maximum variation while minimizing potential bias arising from network-based recruitment, purposive and snowball sampling were combined. The process was conducted in two phases: The interviewer first recruited several interns known to the research team, followed by referrals to additional participants with whom there was no prior relationship, broadening perspectives and enhancing the transferability of findings (39). The study was conducted between June and August 2024. Interviews were carried out face-to-face or via Tencent Meeting on line by the first author (female), ensuring consistency in interview style. All interviews were conducted in Mandarin Chinese; key quotations used in the paper were translated into English and cross-checked by a bilingual team to ensure accuracy. Each session lasted 35–60 min and was audio-recorded with consent and transcribed verbatim. In contrast to studies where observers may be present, all interviews were conducted with only the participant and the researcher present to ensure confidentiality. A single interview was conducted with each participant; in line with the methodology of similar qualitative studies, repeated interviews were not conducted.

Participant recruitment and data collection occurred concurrently until data saturation was achieved. A total of 20 eligible interns were invited to participate. In our study, four of the 20 invited interns declined to participate due to scheduling conflicts, an outcome that is not uncommon in qualitative research involving medical professionals (40). Data collection ceased after the 16th interview when analysis of subsequent interviews yielded no new themes, indicating that data saturation was reached (41). Finally, a total of 16 clinical internship medical students were interviewed, from a regional medical university located in Northwest China. The basic information is shown in Table 1. Research has shown that medical students, both undergraduate and graduate, experience similar stress and adaptation challenges as they transition from classroom learning to clinical practice, including anxiety associated with changing roles, increased workload, and stress from interacting with patients (42–44). Therefore, taking undergraduate and graduate students as a unified research object is helpful in understanding the psychological adaptation process of medical students in practice.

**TABLE 1:** Basic information of interviewed clinical internship medical students.

Interviewee	Age	Major and academic level	Interview method and duration	Code
A1	28	Pharmacy (Undergraduate)	Offline, 50 min	01-ZD
A2	30	Rehabilitation (Undergraduate)	Online, 45 min	02-LY
A3	30	Family medicine (Undergraduate)	Offline, 35 min	03-WX
A4	28	Surgical specialty (Postgraduate)	Online, 40 min	04-LQ
A5	28	Radiology (Undergraduate)	Online, 35 min	05-ZT
A6	31	Radiology (Undergraduate)	Offline, 45 min	06-SF
A7	27	Cardiothoracic surgeon (Undergraduate)	Online, 40 min	07-ZL
A8	27	Ophthalmology (Postgraduate)	Offline, 35 min	08-WM
A9	30	Public health (Undergraduate)	Online, 40 min	09-XX
A10	27	Medical laboratory science (Postgraduate)	Offline, 50 min	10-CY
A11	28	Psysical medicine and rehabilitation (Undergraduate)	Online, 40 min	11-GY
A12	28	Pediatrician (Undergraduate)	Offline, 40 min	12-LM
A13	27	Family medicine (Undergraduate)	Online, 35 min	13-HX
A14	29	Radiology (Undergraduate)	Offline, 35 min	14-GJ
A15	30	Cardiologist (Postgraduate)	Online, 40 min	15-PX
A16	29	Ophthalmology (Undergraduate)	Online, 45 min	16-CF

To protect the privacy of the interviewees, the letters in the codes are randomly chosen and do not represent any meaning.

### 2.7 Interview method and outline

The formation of career resilience of medical students in clinical practice was often reflected in daily events. Flanagan (45) first introduced the Critical Incident Technique (CIT) as a method for collecting direct observations of human behavior that have critical significance in achieving desired outcomes, particularly in educational and professional settings (45).

Initially, the Situation-Task-Action-Result (STAR) framework was used to guide the interview framework (46). The STAR framework, rooted in behavioral interview theory (47), facilitates structured recall of key incidents, enhancing the reliability and validity of behavioral data in competency research (48–50). However, as the study progressed and interactions with interviewees deepened, the interview format gradually shifted toward semi-structured interviews. The questions were adjusted to better align with the emerging themes, evolving into a more flexible approach to allow the participants to share their experiences more freely.

The STAR framework was initially employed to structure the interview questions, but it was supplemented with open-ended questions that followed the principles of CIT, which focuses on gathering detailed descriptions of significant events that shape participants’ resilience (51).

The interview questions were designed to explore several aspects: work situations, professional dilemmas, psychological feelings, behavior choices, external support, and subjective needs (52). This shift to semi-structured interviews enabled the researchers to capture both structured and unstructured data, offering a richer perspective on the participants’ experiences (53). This methodological shift from a structured to a semi-structured format was a deliberate part of the grounded theory process, allowing for deeper exploration of emergent theoretical concepts. To ensure data comparability and consistency across all 16 interviews, the interviewer used the six core domains of the interview guide as a consistent checklist. While follow-up questions varied to probe emergent themes, this core structure ensured that all key topics were systematically covered with every participant, thus maintaining the thematic comparability required for analysis. To ensure that the interviews focused on presenting rather than forcing, the preliminary interview did not communicate with respondents entirely around clear research questions. As the interactions with interviewees deepened, the interviews gradually evolved into semi-structured conversations, allowing the respondents to guide the discussion within the general framework of resilience. The interview was not conducted mechanically and strictly according to the outline order but was adapted based on the participants’ answers. The interview outline is presented in Table 2. The questioning approach consisted of three main types:

1. Supplementary questions to clarify or expand on the events mentioned by interviewees, aiming to restore the full picture of the incident as much as possible.
2. Explanatory questions to clarify native concepts, unfamiliar technical terms, or ambiguous expressions.
3. Clarifying questions to resolve apparent contradictions in the interviewee’s statements or questions that they did not fully understand.

**TABLE 2:** Interview outline design.

Questions types	Technique	Outline or instance
Fundamental questions	Critical Incident Technique (CIT)	1. Working environment: A comprehensive exploration of interns’ background in the medical setting, including their daily clinical duties and the nature of their medical profession.
		2. Professional challenges: Help interns reflect on and discuss any difficulties they encountered during their clinical internship, describing the form of these challenges and their impact.
		3. Psychological responses: Designed to reproduce the true psychological experiences and emotions experienced by interns in difficult clinical situations.
		4. Behavioral responses: Describe in detail how these interns respond to or overcome career problems.
		5. External support: What support interns seek from peers, tutors, or schools when they encounter difficulties.
		6. Subjective needs: Explore the group’s future career development and resilience needs.
Probing questions	Detail-oriented probing	1. Identify the individuals involved in a particular situation. (Who).
		2. Actions taken during a particular medical or clinical procedure (What).
		3. The physical or institutional settings of subsequent events (Where).
		4. Timing and sequence of the events in question (When).
		5. How the event is handled after it occurs (How).
	Interpretive probing	Instances where statements like “a task is unfulfilling” can be unpacked include having to determine if fulfillment for a physician lies along personal interest, financial reward, or other elements; furthermore looking into how this present job tallies with this viewpoint as well as straying away from it could bring out an explanation.
	Clarifying probing	For example, one may ask whether “hours worked beyond normal time” refers back to being called at home on a holiday earlier said before unscheduled duty callings so that his/her story becomes contextualized.

A detailed outline of the interview structure, which integrates the CIT and multiple probing strategies, is presented in this table). This table illustrates how the interview evolved from a STAR-based format to a semi-structured format, enabling in-depth exploration of interns’ career resilience development.

Interviews were conducted by trained members of the research team using a calibrated protocol. The first author, who is trained in qualitative inquiry, kept a reflexive journal to document preconceptions, positionality, and affective reactions across data collection and analysis. Peer-debriefing discussions with two independent qualitative-methods researchers were used to challenge emerging interpretations and enhance analytic rigor. An operational example of the case summary process is presented in Table 3, illustrating how the six-dimensional coding paradigm was applied in this study.

**TABLE 3:** Case summary operation example.

Category	Case summary (code: 03-WX)
Phenomenon	He was enthusiastic about becoming a pediatric intern and earned excellent grades in theory. Confident and motivated, the individual has high professional aspirations and a sense of responsibility. However, he is dissatisfied with the current work climate and feels challenged.
Causal conditions	The working environment is full of pressure; the patient’s family exerts a lot of pressure in communication, and the workload is not proportional to the expected salary of the post. Promotion opportunities were also found to be very limited among the teaching staff, so career prospects were unclear.
Context	He had high career expectations: the job meant continuous personal fulfillment and professional success. In his work, he encountered both competition and cooperation from his peers, and also felt the disconnect between ideal and reality.
Intervention conditions	Although there are many things that make me enjoy this job, such as its high relevance to the expertise I have learned in my life, especially my passion for pediatric medicine, and the positive personality traits of respect and admiration for the medical profession.
Action strategies	Even though professional knowledge matches job responsibilities, people still feel helpless when faced with communication problems or management problems. In this case, he copes with stress by strengthening self-management and emotional regulation, trying to find ways to improve the work environment to increase job satisfaction.
Consequences	He considers the current situation and prepares for graduate examinations to look for opportunities that better meet personal professional growth and self-fulfillment needs. He looks forward to achieving a more balanced work-life state on his career path and gaining more career growth potential in the future, leaving more room for career development.

This table illustrates how raw interview transcripts were distilled into structured analytical categories—phenomenon, causal conditions, context, intervention conditions, action strategies, and consequences—using the six-dimensional coding paradigm, thereby laying the groundwork for open, axial, and selective coding.

### 2.8 Text coding results

Interview transcripts were imported into NVivo software (version 12) for systematic organization and coding. Following Straussian grounded-theory procedures, open, axial, and selective coding were performed iteratively with constant comparison. Regular peer-debriefing sessions were held with two independent researchers experienced in qualitative methods who were not involved in data collection.

#### 2.8.1 Open coding

The coding process followed Corbin and Strauss’s three-step procedure: first, Open Coding using participants’ own phrases verbatim to assign conceptual labels; second, Axial Coding to group causal and intervening relationships into higher-order thematic clusters; and third, Selective Coding to integrate all categories around the core category career resilience development during the role-transition period. This core category became the focal point of our analysis (54). Thus, career resilience development in transition was the preeminent focus of this analysis.

Open coding. Based on trying to use the interviewees’ original words, the interview data of medical students in the clinical practice stage were conceptualized and classified. A total of 226 initial concepts were obtained, and 34 first-level concepts were formed through open coding. For instance, one intern remarked, “I attend the latest medical update courses each week to keep up with rapidly changing guidelines,” which we coded as the initial concept Continual Education. After completing open coding, we proceeded to axial coding to explore relationships among initial concepts.

#### 2.8.2 Axial coding

Axial coding establishes relationships among initial concepts through comparison, leading to the formation of nine higher-order categories along with their attributes and dimensions. For instance, the open codes Technological Adaptation (“Update technical knowledge to maintain clinical competitiveness in the face of complex equipment”) and Continual Education (“Attend medical update courses and respond to rapidly changing guidelines”) were both grouped under the axial category Knowledge Updating. After finalizing these nine categories, we proceeded to selective coding to identify the core category and integrate all others around it.

#### 2.8.3 Selective coding

Selective coding is used to identify the core category and secondary category through systematic analysis and to construct the theoretical framework of the core category based on this. For example, we linked the axial categories Knowledge Updating, Compliance and Pressure Management, and Innovative Breakthrough to form the core category Career Resilience Formation Mechanism, illustrating how these three processes operate in a self-reinforcing loop. During axial and selective encoding, recurring concepts such as adaptive identity, coping beliefs, and peer reinforcement recurred in each case without new categories or attributes. This indicates theoretical saturation, as core dimensions are fully developed without further theoretical insights or category changes. The results of the three-level coding of career resilience of medical students in the clinical practice stage are shown in Table 4.

**TABLE 4:** Coding the results of the career resilience of interns.

Selective coding	Axial coding	Open coding	Initial concept examples (partial)
Career development challenges	Knowledge updating	Technological adaptation	Update technical knowledge to maintain clinical competitiveness in the face of the challenges of operating complex medical devices.
		Continual education	Attend medical update courses and respond to rapidly changing medical guidelines and treatment options.
	Identity establishment	Value exploration	Find personal professional mission and social contribution in daily medical professional activities.
		Role identification	Collaborate with colleagues in different departments to establish your professional status and role in the team.
	Career planning	Goal setting	Develop a career plan that defines career development and promotion goals for the next few years.
		Developmental pathway	Consider a variety of career opportunities, including clinical, teaching or research.
Facilitative resources	Guidance and feedback	Guidance acquisition	Gain clinical experience and plan for the future through exchange programs with experienced doctors.
		Feedback reception	Actively seek advice and feedback from colleagues during team meetings and work processes.
	Resources and opportunities	Training participation	Leverage hospital resources and external training opportunities to enhance professional skills and medical knowledge.
		Material access	Read the latest medical research regularly to get inspiration when treating patients or conducting research.
	Emotional support	Family support	Encourage and support family members at key points in career development.
		Emotional connection	Build trust-based relationships with colleagues and work together to solve problems.
Proactive career adaptation	Compliance and pressure management	Endurance -passive tolerance	Maintain professional communication and patience during night shifts and intense work.
		Compliance-attempted adaptation	In a tense fast-paced environment, learn to relax and adapt to the pace of work.
		Transformation—mindset shift	Empathize with anxiety and turn challenges at work into opportunities for personal growth.
	Innovative breakthrough	Avoidance—crisis reduction	Effectively handle emergency medical tasks and reduce stress levels at work.
		Feedback—active communication	Active communication helps solve problems and share experiences to improve team efficiency.
		Change—environmental modification	Adapt to changes in the medical field by finding new treatments and continuing to learn.
		Compensation -alternative achievement	Innovate in the face of career difficulties and broaden the path of personal career development.

## 3 Research results analysis

### 3.1 Participant characteristics

Sixteen clinical interns (from a regional medical university in Northwest China) participated in the study, including both undergraduate and postgraduate students from multiple specialties. Interviews were conducted online and offline, each lasting approximately 35–50 min (see Table 1).

**FIGURE 1:**
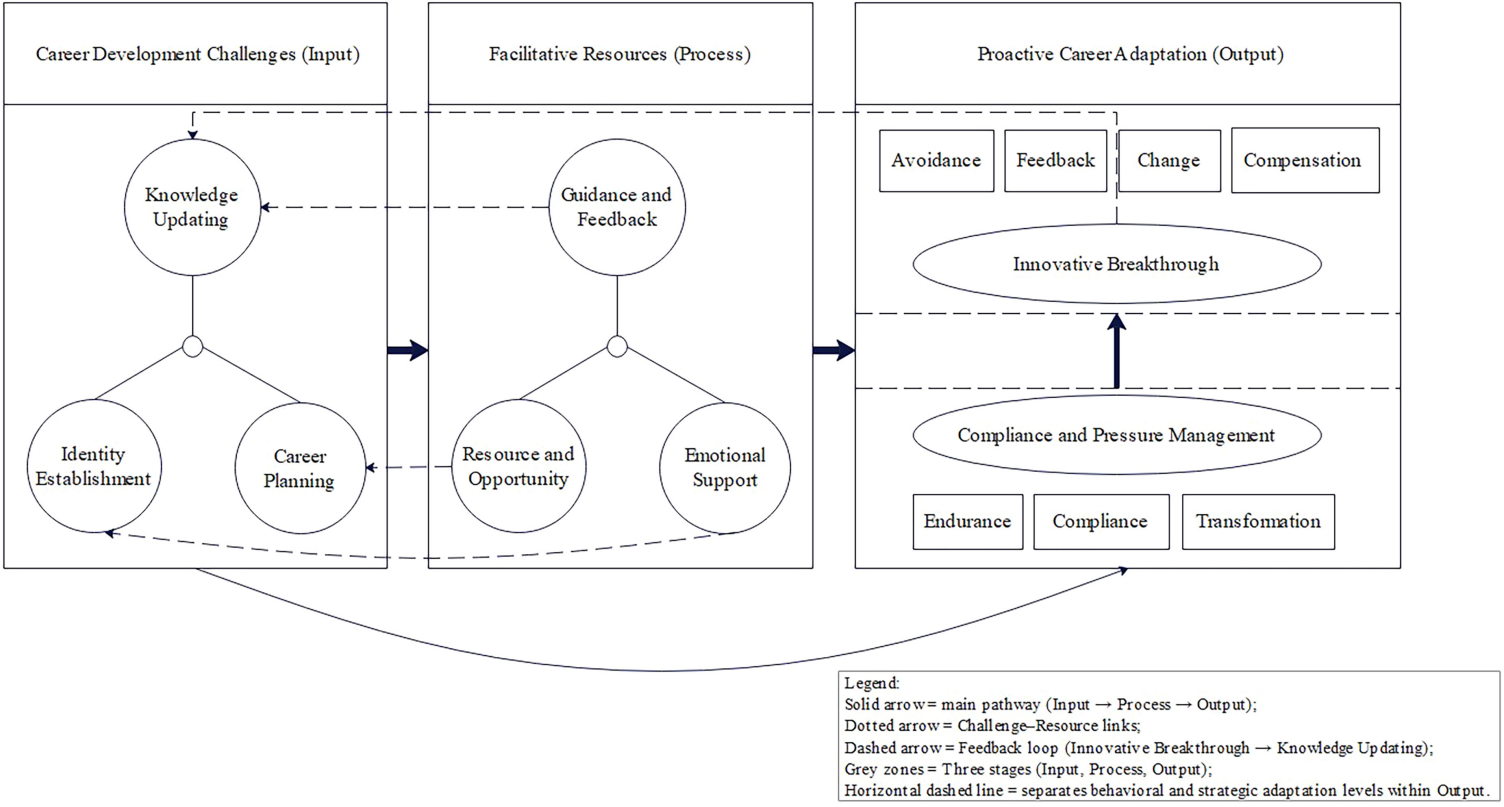
A CRA mechanism model for the formation of the career resilience of interns.

The interview results show that the development of career resilience in clinical interns is embedded in a three-dimensional space constructed by career development challenges, facilitative resources, and proactive career behaviors (see Figure 1). Career development challenges, as an input, are the initiator of career resilience and the precondition of training. Facilitative resources play protective, motivational, or facilitative roles in career development challenges, helping medical students cope with challenges in clinical practice, endowing them with internal motivation, and promoting career resilience. Proactive career behaviors help to transform career development challenges into positive states. This adaptive behavior of turning crisis into opportunity is the explicit form of career resilience transformation of medical students in clinical practice, that is, the so-called “turning stress into motivation.” The next section will discuss the mechanisms that shape the career resilience of medical students in clinical practice in specific clinical settings and service contexts.

The model comprises three sequential stages—Career Development Challenges (Input), Facilitative Resources (Process), and Proactive Career Adaptation (Output)—which operate in a continuous, self-reinforcing loop. Career Development Challenges include Knowledge Updating, Identity Establishment, and Career Planning, which trigger the mobilization of Facilitative Resources such as Guidance and Feedback, Resource and Opportunity, and Emotional Support. These resources enable Proactive Career Adaptation through two progressive levels: the Behavioral Adaptation level (Compliance and Pressure Management), characterized by Endurance, Compliance, and Transformation, and the Strategic Adaptation level (Innovative Breakthrough), manifested through Avoidance, Feedback, Change, and Compensation. Solid arrows indicate the main developmental pathway (Input → Process → Output); dotted arrows represent Challenge–Resource links; and the dashed arrow depicts the feedback loop from Innovative Breakthrough to Knowledge Updating, signifying that each breakthrough generates higher-order challenges and renewed learning cycles.

### 3.2 Initiating career resilience: exploring three major career development challenges

In resilience research, one of the core problems is how individuals adapt behavior, reconstruct cognition, and finally build internal capabilities when facing challenging or stressful situations. In this study, interviews with 16 clinical interns showed that the initial construction of career resilience often began with three Career Development Challenges: Knowledge Updating in rapidly changing clinical situations; Identity Establishment under multiple role relationships and realistic pressures; and Career Planning through rotational practice and career choices. These situations not only constitute the stressors of the internship stage but also become the key nodes to drive individual reflection, adjustment, and growth. Adaptation strategy to ability formation runs through a self-reinforcing spiral process of perception, adaptation, and capability development and is self-reinforcing, which is also the starting point and logical basis of the dynamic mechanism of Career Development Challenges–Facilitative Resources–Proactive Career Adaptation shown in Figure 1.

#### 3.2.1 Knowledge updating: exploring yourself in the dynamic field of internship

At the beginning of clinical practice, many respondents experienced adaptation challenges brought by Knowledge Updating, especially in the process of translating theoretical knowledge into clinical practice. 01-ZD said: “When I looked up the case data, many clinical practices were different from those written in our textbooks, so I didn’t know how to prepare at once.” This theory-context mismatch inspired interns to reassess their learning styles. At first, this gap creates anxiety and discomfort, but most interns do not shy away from the task; but gradually explore alternative paths to meet the challenge. They gradually adapt to the new logic of knowledge application by repeatedly participating in practical activities such as clinical case discussion, virtual surgery training, and department rotation. 02-LY said: “When I first did VR surgery, I found that I didn’t know anything, but I especially wanted to understand it and was willing to ask more and learn more.” In this process, interns not only strengthen their operational skills, but also gradually form the ability to reflect and adjust. They are able to anticipate gaps in their knowledge, consciously seek out Supplementary Information, reexamine initial gaps, and turn them into motivation for learning. This evolutionary path from perception gap to self-drive constitutes a key link in the construction of career resilience. In the clinical context, Knowledge Updating is no longer static knowledge reception, but a circular process in which interns reshape professional understanding continuously in dynamic feedback with the help of intrinsic motivation and external support, and then activate Proactive Career Adaptation. PG 08-WM emphasized how rapidly changing clinical technologies required immediate mentor-guided practice:“In our department, new imaging technologies and surgical techniques change extremely fast. Sometimes I’m introduced to a new concept in the morning, and by the afternoon my supervisor will focus on it during demonstration and ask us to practice repeatedly in the skills center. This way, by the next day I can often perform a specific step of the surgery independently.” Across cases, a recurrent sequence emerged whereby a mismatch between practice and textbook knowledge elicited mentor-guided rehearsal or checklisting, which in turn led to a discrete behavioral upgrade.

#### 3.2.2 Identity establishment: the construction of the “I” in professional relationships

For medical students in the early stage of internship, entering the hospital is not only a task change but also a process of Identity Establishment. The original role of the student is constantly challenged and reconstructed in real interaction, especially under the multiple expectations of tutor, patient and peer. Interns gradually feel the sense of professional responsibility. 03-WX pointed out: “The teacher taught me surgical skills while emphasizing communication with patients and ethical judgment, and then I really realized that I would become a doctor.” In diverse department rotation and cross-specialty collaboration, interns gradually adjust their behavior orientation through case discussion, collaborative operation and daily feedback, and modify their professional expression in the interactive experience. 02-LY recalls: “In the face of complex cases, we began to communicate like doctors, not just students memorizing knowledge points.” Some respondents mentioned that due to emotional fluctuations caused by doctor-patient interaction, guidance evaluation, or unexpected situations, they would actively reflect on coping styles and adjust behavior boundaries and communication strategies in subsequent practice. This process evolves through successive role-plays, value judgments, and situational feedback. From the initial establishment of role cognition to the strengthening of value preference, interns gradually embed self-orientation into professional situations and form behavior patterns that cooperate with professional norms. Finally, Identity Establishment becomes a continuous adaptation process, which embodies the evolution logic from cognitive understanding, and behavior adjustment to ability internalization in the doctor-patient relationship and teaching interaction, laying a foundation for the subsequent development of career resilience. (04-LQ) explained how professional identity was shaped through emotionally charged encounters and supervisory reflection: “Once I was questioned simultaneously by a patient’s family and a senior doctor; I felt very passive and frustrated. After the operation, my supervisor spent time reviewing the whole situation with me and the team, analyzing what went wrong in the communication. Since then, when facing similar situations, I’ve been much steadier about where the boundaries are and how to phrase my responses.” This reflects a pattern whereby stressful interactions with patients or supervisors led interns to seek supervisory feedback and team discussion, which then fostered greater confidence and clearer professional boundaries in subsequent communication. Exploratorily, undergraduates tended to describe “speaking like a doctor” for the first time, whereas postgraduates more often emphasized boundary management and emotional regulation in complex interpersonal contexts.

#### 3.2.3 Career planning: shaping self through personal vision and career reality

For most clinical interns, Career Planning is not a one-time decision-making behavior, but a gradual process of continuously connecting internal expectations with real experience in department rotation. 03-WX said: “The pediatric experience made me realize that I like dealing with children very much, and made me start thinking about whether I can develop in this direction in the future.” These tendencies do not arise instantaneously, but gradually develop over a continuous experience of different job content, patient characteristics, and organizational climate. As clinical tasks enrich and exposure expands, interns constantly reassess their interest preferences, role suitability and long-term development potential, thus accumulating adjustment experience in situational feedback. Some students gradually transform others’ experiences into their own judgment basis, showing strong initiative. 01-ZD: “When listening to experts talk about future development, I will seriously consider whether to take the exam first or work first.” This reflective behavior reflects an experience-driven capacity for self-regulation. Interns no longer passively accept path suggestions, but form judgment frames and action strategies that match clinical situations in repeated exploration and evaluation. This process unfolds from situational triggers through subjective evaluation to behavioral transformation, driving the construction of the three-dimensional mechanism of Career Development Challenges-Facilitative Resources-Proactive Career Adaptation to navigate career uncertainty. Finally, Career Planning not only focuses on the definition of goal direction but also reflects the career resilience development track of interns, integrating internal and external resources and continuously adjusting in complex situations. (08-WM) further reflected on how critical clinical exposure shaped long-term orientation: “The first time I observed macular surgery under a high-magnification microscope, the precision and challenge fascinated me. From that moment, I basically decided to specialize in retinal diseases.” (10-CY) revealed that professional planning was often refined through cyclical practice and reflection: “After I finally mastered a new protocol, I was soon assigned to a more difficult case, which immediately made me aware of new knowledge gaps. I had to start another round of study and updating.” This illustrates a pattern whereby advanced exposure to complex tasks and technologies led interns to clarify career directions and form specific plans for further study, as repeated practice and feedback deepened their self-awareness and commitment to chosen paths. Exploratorily, undergraduates more often described Career Planning in terms of task enjoyment and patient-group preference, whereas postgraduates linked it to subspecialty or research trajectories developed through advanced clinical exposure. This mechanism, together with the Knowledge Updating and Identity Establishment mentioned above, constitutes the three-dimensional development structure of the clinical practice stage and becomes an important psychological foundation for adapting to future career paths. Taken together, challenges in Knowledge Updating, Identity Establishment, and Career Planning constitute the three core career development challenges confronting clinical interns and serve as the initiating inputs in the CRA model of career resilience formation.

### 3.3 Hatching career resilience: three-dimensional facilitating resources

During internships, medical students mobilize Facilitative Resources to buffer Career Development Challenges. These resources form a key fulcrum for managing stress and reinforcing adaptive beliefs. Interviews show that Facilitative Resources can be classified into Guidance and Feedback, Resource and Opportunity, and Emotional Support. These Facilitative Resources are internalized into driving forces that underpin Proactive Career Adaptation during interns’ cycles of reflection and learning. Together, they establish a foundational platform enabling medical students to navigate Career Development Challenges and the role-transition period, thereby activating the CRA model of career resilience formation. The following will be divided into dimensions to reveal how the three types of resources work together to support medical students in moving toward self-confidence and responsibility in practice.

#### 3.3.1 Guidance and feedback

Clinical Guidance and Feedback are often seen as important pivots at the beginning of the internship, helping interns familiarize themselves with the clinical environment and initiate Proactive Career Adaptation. Many interviewees mentioned that clinical supervisors not only helped them familiarize themselves with the internship process, but also provided experiential guidance at key nodes such as emergency response and operational specifications. As (03-WX) said: “The teacher first familiarized me with the process and let me participate in emergency treatment. That experience trained many skills and made me more confident in myself.” With the deepening of practice, the role of clinical supervisors gradually shifts from operation guidance to corrective feedback on critical tasks, helping interns identify shortcomings and adjust behaviors. (05-ZT) mentioned that after sharp but sincere comments from his mentor, he realized the weak link in the surgical operation and made system improvements. The value of this feedback is not limited to technical aspects, but also to stimulate self-efficacy and clinical safety in interns. Over successive stages, from orientation to skill refinement to behavioral internalization, mentorship evolves and embeds key practices into interns’ professional schema. This staged integration of Guidance and Feedback catalyzes career resilience development. (15-PX) explained how immediate feedback during high-pressure procedures accelerated his skill refinement and confidence: “Once, during an operation, the director pointed out that my key hand movement was wrong and demanded immediate correction. That evening, I re-practiced on the simulator and completely revised my technique. The next day, after seeing my improvement, the director allowed me to take on a more complex task.” This illustrates how timely, specific feedback prompted interns to revise their operational strategies and re-engage with higher-level tasks, transforming corrective supervision into Facilitative Resources for growth and reinforcing adaptive professional confidence.

#### 3.3.2 Resource and opportunity

Interns often perceive access to Resource and Opportunity as fundamental to their growth and drives related to the internship. Participant (09-XX) recalled the following: “Having advanced equipment that I cannot access in school makes me feel like I’m growing up fast and provides a stronger motivation for me to study.” In the initial phases, exposure to sophisticated clinical tools and new technologies was a source of novelty that students actively engaged with. The various departments had different learning opportunities, which included specialized procedures, up-to-date teaching materials, and participation in multi-disciplinary teams, all of which enhanced the application of learned concepts and contextual understanding over time. Most importantly, these material resources were not independent of one another. As participants highlighted, pertinent clinical opportunities like complex tasks and professional dialogue made accessible these resources. These opportunities helped interns see themselves as valuable contributors. They fostered greater confidence, willingness to explore, and flexibility in the face of clinical uncertainties. In this manner, the effect of resourcefulness and opportunity was not limited to one phase but developed further as a progressive catalyst. Beginning from outer stimuli and accessibility, interns incorporated these exposures into a broader developmental trajectory, where they had the ability to deeply and persistently engage with the field, accompanied by the co-evolution of competence and resilience. (08-WM) noted that exposure to advanced facilities and complex operations strengthened her professional commitment: “When I got to use the department’s advanced equipment and assist in complex surgeries, I realized how much I liked this subspecialty. I even asked to join more operations and look for extra training opportunities.” (07-ZL) expressed a similar sense of motivation derived from hands-on exposure: “When I was finally allowed to handle new instruments and join the discussion of tough cases, I felt much more driven to learn. After that, I started setting extra practice goals for myself.” Taken together, these accounts illustrate how concrete access to advanced technologies and challenging cases encouraged both exploration and long-term commitment, demonstrating how Resource and Opportunity function as Facilitative Resources that foster Proactive Career Adaptation. Opportunity exposure gave interns a sense of professional belonging and direction, which in turn fostered greater persistence and proactive engagement in learning. Such access to Resource and Opportunity, therefore, contributes directly to interns’ career resilience development.

Below, we turn to emotional support as our third resource dimension.

#### 3.3.3 Emotional support

In a clinical environment with heavy responsibilities and high stress, Emotional Support has become an indispensable buffer mechanism for clinical interns. In the early stages, many interviewees relied on peer support to relieve stress and understand each other. For example, (13-HX) stated, “Weekly meals with classmates relieve stress and we understand each other’s clinical difficulties.” As the internship progresses, the forms of emotional support evolve. Some interns will actively adjust the pace of life to achieve physical and mental balance. As stated in (14-GJ): “Going out on weekends helps me get better at work on Mondays.” In addition to individual and peer support, some hospitals offer structured services such as psychological counseling and stress relief lectures. When the instructor or manager demonstrated an empathetic understanding of the interns’ emotional states, such as providing flexibility in high-pressure situations, this organizational-level Emotional Support significantly enhanced the interns’ sense of belonging and responsibility. On the whole, emotional support does not exist statically, but progresses continuously in different stages, from peer support to self-adjustment, then to institutional care, to construct the Emotional Support gradient of career resilience formation. (15-PX) mentioned a more formal source of support that helped him manage stress and return to work: “There was a time when I felt so overwhelmed that I almost wanted to quit. Then I tried the hospital’s counseling hotline for residents, and my mentor also adjusted my schedule a bit. After taking a short break, I calmed down and was able to focus again.” This shows how institutional and supervisory care turned moments of emotional exhaustion into opportunities for recovery and renewed engagement, illustrating how Emotional Support functions as a Facilitative Resource within the CRA model to sustain Proactive Career Adaptation. When structured support complemented personal coping, interns were better able to regain focus and maintain motivation in high-pressure settings. Initially, undergraduates more often relied on informal peer venting, while postgraduates were more likely to seek formal channels such as counseling or flexible scheduling.

#### 3.3.4 Linking facilitative resources to career challenges

This section describes how three types of Facilitative Resources cushion three major Career Development Challenges. First, Guidance and Feedback bridge the Knowledge Updating gap. (01-ZD) shared: “The tutor will take us to learn the latest medication guidelines every week, and also arrange to practice dispensing in the skill room. The next day, I was able to perform the ward rounds on my own. I felt that I could make up for the gap in a short time. It was very reassuring.” (07-ZL) shared, “When we simulate new equipment in the skill center, the teacher will demonstrate it first, then let me practice it myself, and finally watch the video together to find out the details. After practicing a few times, I was basically familiar with it.” Thus, structured feedback and hands-on demonstrations are key pathways linking Knowledge Updating and Guidance and Feedback; Second, Resource and Opportunity help clarify the uncertainty of Career Planning. Resource and Opportunity, such as advanced equipment, interdisciplinary rotation and industry expert lectures can effectively reduce the pressure brought by “career choice uncertainty”; Finally, Emotional Support plays a buffer role in Identity Establishment. (13-HX) recalled “After the night shift, I went to eat noodles with my roommate and complained about the embarrassing things of the day. I immediately felt that I was not alone in carrying the pressure. I felt much more at ease.” (06-SF) stated “The first time I was questioned by my family, I collapsed. Fortunately, I called the hotline of the psychological center of the academy and calmed down after talking to the teacher for more than 10 min.” Peer support and formal psychological services can reduce self-doubt in stressful environments and provide emotional energy for subsequent innovative adaptation. Guidance and Feedback mainly supplement knowledge, Resource and Opportunity mainly guide direction, and Emotional Support mainly buffer identity pressure; together they contribute to the career-resilience cycle from Compliance and Pressure Management to Innovative Breakthrough. Taken together, these findings highlight how different types of facilitative resources corresponded to distinct developmental challenges across training levels. Guidance and feedback supported interns in bridging knowledge gaps and improving technical confidence (e.g., 01-ZD, 07-ZL), while access to advanced opportunities guided career planning and strengthened long-term direction (e.g., 08-WM, 10-CY). Emotional support, ranging from peer conversations to institutional counseling, helped interns manage identity-related pressure and maintain engagement under stress (e.g., 13-HX, 06-SF). Viewed collectively, these pairings demonstrate that targeted Facilitative Resources addressed specific Career Development Challenges, enabling interns to transform stress into growth and reinforcing the dynamic balance between challenge and adaptation within the CRA model. After the targeted buffering provided by Guidance and Feedback, Resource and Opportunity, and Emotional Support has brought stress back to a manageable level, interns begin to show a two-stage pattern of Proactive Career Adaptation.

### 3.4 Developing career resilience: proactive adaptive behavior

Empowered by Guidance and Feedback, Resource and Opportunity, and Emotional Support, interns first engage in Compliance and Pressure Management; as experience accumulates, they progressively shift toward Innovative Breakthrough. In the process of clinical practice, medical students do not just passively bear pressure, but gradually internalize external challenges into career resilience in continuous practice. Early on, they tend to maintain basic working conditions by obeying rules and restraining emotions to avoid mistakes or conflicts. Although these coping strategies can stabilize the situation, they are mostly short-term effective and difficult to support deep-seated professional growth. As the internship progresses, some students reflect on their interactions with patients, colleagues, and mentors, gradually adjust their own rhythm and behavior strategies, and begin to actively deal with complex problems at work. They gradually develop more flexible modes of action, drawing on peer experience, mentor advice, or situational observation. This change reflects the transformation from passive coping to Proactive Career Adaptation, and also marks the gradual incubation and evolution of career resilience in concrete practice. This section focuses on two coping paths, namely Compliance and Pressure Management and Innovative Breakthrough, and further illustrates how interns develop career-resilient behaviors according to their own characteristics at different stages.

#### 3.4.1 Compliance and pressure management

Facing the high-pressure and high-regulation clinical environment, many interns choose obedience and forbearance as the main coping methods at the initial stage. As interviewees (01-ZD) pute it: “Hospital rules are strict and we have to follow them to avoid unnecessary conflicts and mistakes.” (02-LY) added: “Night shifts are so exhausting — you just grit your teeth and get through them.” This strategy appears mainly during the early phase of clinical practice. Interns are still in the adjustment period of department rhythm, power structure, and clinical norms. By obeying the system and suppressing emotions, although short-term stability can be maintained, it is not enough to support subsequent professional growth. With the accumulation of work experience and the improvement of situational familiarity, some interns gradually realize the limitations of passive adaptation and begin to reflect on how to allocate energy more reasonably and adjust expectations. This change indicates a potential shift from single obedience to positive adaptation, and obedience and repression thus become transitional states in the development of career resilience. (04-LQ) described this stage: “At that time, I had to follow every step exactly and even hold back my own feelings or ideas. It did help me avoid mistakes for a while, but I also felt my growth was limited until I gained more confidence and support to change the way I worked.” This shows how strict clinical routines and hierarchical expectations initially pushed interns toward rule-bound coping. Such discipline maintained stability but offered only temporary control, prompting reflection on how to adjust once confidence and supportive resources became available.

#### 3.4.2 Innovative breakthrough

On the basis of reflecting on the feedback from tutors, the interns gradually formed their own strategies and behavioral preferences to deal with problems by constantly adjusting their clinical operation methods. As described in (05-ZT): “After the teacher pointed out the problems in my operation, I took the initiative to find ways to improve, and my operating ability improved gradually.” (06-SF) also said: “Flexibility is needed to respond faster and better to different patient needs.” These changes reflect the self-adjustment and creativity of interns under limited conditions. They no longer only passively adapt to the environment, but actively seek improvement paths and accumulate self-efficacy and a sense of achievement in trial and error. The transition from obedience to active exploration is the process of gradual construction of career resilience. In the short term, it depends on adaptation to maintain operation, and in the long term, it depends on continuous optimization to achieve growth. In multiple challenges, medical students gradually develop the ability to cope with uncertainty and shape stable and flexible career resilience in action. (11-GY) recalled, “The moment I finally nailed that new treatment protocol, my supervisor handed me an even tougher case. Every time I level up, the game dials up the difficulty again.” 10-CY reflected on how progress in one stage often triggered a new round of challenges: “After I finally mastered a new protocol, I was soon given a more difficult case. That immediately made me realize new gaps in my knowledge, so I had to start another round of learning and updating.” This suggests that each Innovative Breakthrough is followed by rising task difficulty, exposing further areas for improvement and prompting renewed Knowledge Updating and skill refinement. Continuous feedback between success and renewed challenge thus kept the cycle of adaptation and learning in motion, showing how innovation itself became the driver of resilience growth. Such reflections show that every breakthrough quickly elevates performance requirements. Once an Innovative Breakthrough generates higher-order tasks such as a new round of knowledge updating, it sets in motion the next CRA cycle of Career Development Challenges, Facilitative Resource mobilization, and Proactive Career Adaptation. Exploratorily, postgraduates more often reported earlier transitions from compliance to innovation when rotations afforded higher-complexity tasks and tutor-granted autonomy, whereas undergraduates tended to remain in compliance longer during initial acclimatization.

## 4 Discussion

The findings of this study reveal that career resilience among medical interns is a dynamic, relational, and developmental process rather than a fixed individual trait. Through the interaction of career development challenges, facilitative resources, and adaptive behaviors, interns construct resilience as they navigate the high-pressure, uncertain environment of clinical training. The proposed Challenge–Resource–Adaptation (CRA) model illustrates how specific clinical challenges—such as knowledge updating, identity formation, and career planning—activate the mobilization of resources, including mentor feedback, learning opportunities, and emotional support. These resources, in turn, foster adaptive behavioral responses ranging from compliance and pressure management to innovative breakthroughs. Over time, repeated challenge–response cycles enable interns to transform stress into growth, gradually forming a self-reinforcing loop of professional adaptation and learning.

By integrating micro-level personal experiences with institutional and cultural contexts, the CRA model reframes career resilience as a context-embedded learning cycle rather than a simple “bounce-back” reaction. This perspective provides a bridge between individual psychological adaptation and systemic educational design, offering a more process-oriented understanding of how resilience is built, sustained, and transmitted within medical training environments. Through case studies, theory-based methods, and extensive interviews, this study explored the process of career resilience formation in medical students during the transition period of clinical practice.

### 4.1 Career resilience triggered by career development challenges

This study found that clinical interns’ career resilience was particularly prominent when facing challenges such as hospital professional environment, role identity change, and high-pressure task load. Research has shown that career resilience is generally more likely to be activated and exerted only in situations of adversity or high-intensity challenge (55, 56). This finding echoes the findings of interviews conducted in this study: Medical students’ career resilience is not spontaneously generated in situations such as intense night shifts, complex patient coping, or frequent knowledge updates, but gradually emerges and develops in response to real-world challenges. Related studies further suggest that medical interns’ career resilience levels exhibit dynamic fluctuations rather than fixed traits when faced with high uncertainty and emotional load (4). In addition, research has shown that continued exposure to high-stress environments, such as high-risk operations, ethical dilemmas, and team collaboration conflicts, are important triggers for interns to build career resilience (57). Therefore, the challenging environment in clinical practice is not only the source of perceived stress but also the key starting point for the gradual awakening and shaping of medical students’ career resilience. In China’s high power distance medical culture, interns are socialized to unquestioningly follow supervisors’ directives, a pattern that is reinforced by hierarchical norms, so that their initial coping consists primarily of compliance with authority (58). Only when supervisors provide sufficient feedback and grant genuine autonomy do interns feel enabled to move beyond compliance toward exploratory innovation (59, 60).

### 4.2 Identity establishment and patient-centered care

This study indicates that the Identity Establishment of medical students plays an important role in building a professional sense of belonging and coping skills during clinical practice. The formation of identity is not a one-way process. Medical students not only need to master the professional skills of interacting with patients but also must balance the requirements of ethical communication and humanistic care in practice. Patient-Centered Care functions as a facilitative process that strengthens interns’ professional identity (61). In interviews, we observed that medical interns gradually strengthened their sense of identity with the role of doctors as they understood and practiced patient-centered concepts, which made it easier to maintain internal stability and empathy in stressful situations, thus improving their career resilience to challenges.

This finding is supported by recent empirical research. For example, recent research has found that embedding patient perspectives in clinical teaching significantly enhances trainee medical students’ identification with doctors and fosters the development of interpersonal communication and empathy skills (62). At the same time, some studies have further pointed out that medical students will be more active in professional identity construction during multiple interactions with patients and participation in decision-making processes, reflecting the dynamic coupling mechanism between identity and situational experience (63). Moreover, under China’s pervasive face-saving ethos, interns initially avoid risk-taking to prevent loss of face before peers and mentors; once they receive affirmative feedback in a trusting team climate, concern for preserving face can itself motivate reputation-enhancing innovation (64). By linking Identity Establishment to stress adaptation, our findings extend existing work on patient-centered clinical experiences and offer new empirical support for the CRA model of career resilience formation.

### 4.3 Continuous knowledge updating and clinical judgment

In clinical practice, medical students must continuously update their knowledge to keep pace with evolving technologies and standards. This adaptability underpins their developing career resilience. Interviews in this study showed that tutors effectively promoted students’ clinical thinking development and judgment, a key adaptive career behavior, through operational demonstrations, case discussions, and immediate feedback (65). This finding complements existing research on the critical role of mentor instruction in activating clinical judgment (66) and suggests that continuous feedback mechanisms are important pathways for knowledge updating and professional growth in medical students.

In addition, the application of AI–based decision support tools in clinical teaching also provides dual support for medical students to update knowledge and cultivate judgment, helping them to cope with complex tasks more efficiently and enhancing professional confidence and career resilience (67). This study shows how instructional guidance and technical empowerment together facilitate ongoing knowledge updating and clinical judgment, providing practical insights for the dynamic formation of career resilience during internships.

### 4.4 Resources and opportunities: interdisciplinary experiences

Interdisciplinary rotations and exposure to advanced diagnostic technologies broaden medical interns’ clinical perspectives and enhance decision-making in complex patient cases. Such experiences cultivate adaptability, collaboration, and professional confidence by situating learning within diverse disciplinary and institutional contexts (68, 69). Building on earlier discussions of mentorship and feedback, these opportunities further extend the learning process beyond single-department routines, allowing interns to integrate multiple perspectives and problem-solving approaches. The combination of collective teamwork and autonomy nurtures both competence and creativity, reinforcing interns’ capacity to translate complex challenges into collaborative solutions. In this sense, resource-rich and interdisciplinary environments provide the material and social foundations that sustain career resilience and continuous professional growth (70–72).

### 4.5 Emotional support and wellbeing

Medical students interviewed repeatedly mentioned the value of emotional support. Having dinner with peers, sharing stresses with peers, or receiving access to psychological counseling significantly reduced burnout or isolation, thus stabilizing their confidence in continuing to engage in clinical challenges. A number of papers have noted that peer support is critical to reducing nursing burnout and isolation (73) and that providing mental health resources (such as counseling services or stress management lectures) helps clinical interns better cope with mood swings in their professional environment (74). In addition, compassionate leadership enables medical students to feel understanding and tolerance from their superiors, resulting in higher job satisfaction and emotional resilience (75). These literature findings and our results reflect that peer support and compassionate leadership have the same positive effect on the formation of interns’ career resilience. Further, Puranitee et al. (76) emphasized that belonging plays a key role in reducing job burnout and promoting job engagement (76). They found that interventions in medical education that promote a sense of belonging and peer interaction help enhance students’ career resilience. This enhanced emotional resilience underpins broader career resilience development, demonstrating that supportive peer networks and compassionate leadership are key facilitators of interns’ career resilience.

### 4.6 Balancing compliance and innovation: a two-path adaptation model

Interns initially rely on Compliance and Pressure Management, following rules and enduring stress to stabilize emotions and prevent errors. True career growth, however, depends on Innovative Breakthrough, which the model defines through four strategies: Avoidance, Feedback, Changes, and Compensation. When practitioners encounter more persistent or structural challenges, relying solely on grit persistence and patience cannot fundamentally relieve stress, and strategies of frustration tolerance and resilience should be incorporated (Achieving Long-term Goals Amidst Uncertainty: An Integrative Model for the Psychological Resources of Grit, n.d.), combined with creative thinking and feedback loops (77). This also echoes the discussion of (78, 79) on the efficiency and scientization of medical management, through the dynamic adjustment of resources, personnel, and teaching mode. Interns not only relieve the pressure of current tasks but also form continuous upward career resilience. Especially under the interweaving of factors such as current medical service demand, technological progress, and economic pressure, interns can show more outstanding adaptability if they can apply their knowledge in creative ways and flexibly respond to changes in the external environment (80, 81). It can be seen that active Innovative Breakthrough is not optional, but a key path to maintain career resilience and meet greater challenges in the future. Studies have shown that incorporating innovative resilience skills training courses into medical curricula can effectively improve students’ self-regulation and coping skills in complex situations (82). In addition, some literatures indicate that career resilience during clinical practice has an important protective effect on relieving training stress and improving professional life quality (4). Crucially, a climate of psychological safety—where mistakes are not punished—serves as the essential condition enabling interns to move from compliance to innovation and fueling each cycle of the CRA spiral (83).

This internal shift from passive compliance toward active innovation can be further explained by integrating perspectives from Social Cognitive Theory and Self-Determination Theory. Social Cognitive Theory holds that interns raise their innovation self-efficacy by observing mentors, integrating constructive feedback, and internalizing successful practices (84, 85). Once interns’ needs for autonomy, competence, and relatedness are met in a supportive environment, they shift from compliance to intrinsic innovation (86, 87). Thus, the synergy of enhanced self-efficacy (social cognitive perspective) and fulfillment of intrinsic psychological needs (self-determination perspective) explains why interns transition from compliance to Innovative Breakthrough.

### 4.7 Career resilience emerges through a self-reinforcing loop

Resilience has been variously described as recovery, balance, and adaptive growth. Foundational models view it as a return to biopsychosocial equilibrium following disruption, guided by protective factors that enable “reintegration” at or above baseline (28). Process-oriented perspectives emphasize the interaction of personal assets and cognitive appraisals that shape coping responses under pressure (30), while medical-education research depicts a dynamic “reservoir” in which mentorship, social support, and self-care replenish capacity against stress (88). Later frameworks such as the Resilience Activation Framework (89) and the ART model (90) stress iterative adaptation, viewing access to social and emotional resources as the key to sustained recovery. More recent educational models, including the Systematic Assessment for Resilience (SAR) framework (27), operationalize resilience through formative assessment and growth-feedback loops.

The CRA model proposed here extends these perspectives by illustrating a recursive learning cycle specific to early-career role transitions. Rather than depicting resilience as a “bounce-back,” CRA explains how concrete career challenges activate resource mobilization and adaptive behavior that, in turn, generate higher-order challenges and continued development. This context-embedded mechanism integrates mentorship, institutional feedback, and peer networks within hierarchical medical environments, providing a process-oriented explanation of how resilience is actively constructed rather than passively restored. This recursive logic demonstrates how career resilience emerges through a self-reinforcing cycle of challenge, resource mobilization, and adaptive growth, as conceptualized in the CRA framework.

These findings echo recent Chinese and cross-regional studies emphasizing that the interplay between structural hierarchy, institutional resources, and mentoring cultures determines how resilience mechanisms operate (91, 92).

In comparison with existing resilience frameworks, the present findings should be considered within the study’s methodological boundaries. Because the sample was limited to a single institution and derived from purposive and snowball recruitment, the findings represent theoretical generalization rather than population-level inference. As noted in other grounded-theory research on medical trainees, small and context-bound samples are common in early-stage model construction (93), yet they inherently constrain external transferability. Furthermore, the focus on interns midway through their placements captures a critical transition stage but omits longer-term resilience trajectories; consequently, the CRA model presently reflects an early-career mechanism rather than a full professional lifecycle (94). Recognizing these constraints clarifies that our conclusions pertain primarily to conceptual explanation, identifying how challenge, resource, and adaptation interact, while subsequent multi-site, longitudinal, and mixed-method studies are required to confirm the model’s broader applicability.

Likewise, this study highlights that hierarchical and collectivist learning cultures profoundly influence how medical trainees mobilize support and demonstrate adaptation. In Asian clinical settings, steep authority gradients and face-saving norms often channel responses toward rule-bound coping and deference to seniors, while also discouraging help-seeking when distress carries stigma (62, 76). Conversely, mentor guidance, cohesive peer relations, and compassionate leadership consistently buffer strain and sustain engagement in high-pressure environments (73–75). These cross-cultural patterns reinforce the CRA model’s central mechanism of balancing challenge with resource access while underscoring that its implementation must be calibrated to local learning climates so that institutional hierarchy, feedback norms, and social networks act as enablers rather than inhibitors of adaptive growth.

The findings suggest that the development of career resilience can be strengthened through daily educational practices that balance challenge, support, and reflection. In clinical training, progressive exposure to responsibility and authentic patient care seems to stimulate adaptive motivation, especially when combined with clear guidance and formative feedback from supervisors. Mentorship and peer collaboration appear to provide the emotional safety and informational scaffolding that allow interns to translate pressure into purposeful learning. Access to interdisciplinary rotations and simulation-based experiences may further broaden clinical perspectives and confidence, while consistent opportunities for reflective discussion help students connect each challenge to its learning value. When these elements converge, clinical education evolves into a setting where stress functions not as a threat but as a resource for growth, and resilience becomes an integral part of professional identity formation.

## 5 Limitations of the study

This study has the following limitations. First, although our data come from only 16 interns at a single institution, limiting broad generalizability, this uniform setting serves as a coherent cultural lens, revealing how the CRA mechanism functions within a high power-distance and reputation-focused cultural context and offering a benchmark for future cross-cultural comparisons. Although purposive and snowball sampling were applied to broaden participant diversity, this network-based recruitment strategy may still have introduced selection bias and limited the heterogeneity of perspectives. Interns who were more closely connected to institutional networks or more interested in career-resilience topics could have been over-represented, which may constrain the transferability of the findings. Second, by interviewing interns at the midpoint of their clinical placements, a pivotal transition from student to practitioner, we captured the dynamic emergence of career resilience; however, this focus also means that later stages of resilience evolution, including post-internship trajectories, remain to be explored in longitudinal research. Third, the study did not make the cross-specialty comparison of specific professional differences (such as surgery, internal medicine, pediatrics, oral cavity, etc.), and the challenges and resource needs of interns in different specialties may be different. Our interviews were conducted by researchers with clinical experience, a dual role that fostered participant trust and depth of disclosure while also introducing potential expectancy bias. This researcher–practitioner dynamic provides unique insight into the co-constructive process of career resilience formation.

## 6 Conclusion

In conclusion, this study elucidates how medical interns develop career resilience during the transition from student to practitioner. Drawing on grounded qualitative analysis, it identifies that resilience is triggered by transitional challenges, supported by facilitative resources, and manifested through adaptive behaviors that together form a recursive cycle of growth. The proposed CRA model captures this dynamic interplay, showing that resilience is not a fixed trait but an evolving process of learning through adversity. By clarifying how challenges, resources, and adaptive strategies interact to sustain professional development, this study provides an integrated explanation of career resilience formation within clinical education. Based on these findings, the CRA framework can be integrated into routine medical training. Programs should incorporate communication, teamwork, and emotion-regulation as explicit learning outcomes and embed brief challenge–resource–reflection cycles within placements. Mentorship and institutional support can be strengthened through regular debriefs, structured autonomy, reliable skills-lab access, cross-specialty rotations, and timely counseling services. Future research should examine the CRA model across diverse sites and specialties using longitudinal or mixed-methods designs, explore how the frequency and quality of CRA cycles relate to competence and wellbeing, and evaluate moderating factors such as mentoring style and program resources. Intervention studies may further validate CRA-informed approaches and develop portfolio-based indicators to support large-scale implementation.

## Data Availability

The raw data supporting the conclusions of this article will be made available by the authors, without undue reservation.
