# Supplementary material for: Mechanism of career resilience formation during the role transition of medical interns: a grounded theory study
Source: Front Med (Lausanne). 2025 Nov 17;12:1671520. doi: 10.3389/fmed.2025.1671520 (PMC12665529; doi:10.3389/fmed.2025.1671520)
Supplement: Supplementary file 1 [file Table_1.doc]

**Supplementary File S1: Interview Guide**

1. Working Environment

1.1 Fixed Question:

Can you describe your own work during your intership, particularly outlining your clinical responsibilities and the profession in general? What was your work setting like?

1.2 Follow-Up Questions:

Can you describe a typical day in your current role? When you were working, how did this differ from what you had imagined beforehand?

What difficulties did you experience in being comfortable in this working condition?

How do you manage the large amounts of new information in your current role? What strategies or measures have you used to update and improve your skills and knowledge?

Considering the change from a medical student to a intern, what identity challenges have you faced, and how did you manage the balance between your professional self and personal self?

2. Professional/Personal Aspects of Medical Projects

2.1 Fixed Question:

Can you describe any difficulties you personally faced while working on medical projects? What specific problems interfered with your work?

2.2 Follow-Up Questions:

I would be particularly grateful if you could give me one instance of a medical project in which the developments occurred differently from what had been planned. What do you think were the causes of the difficulty?

What were your emotional responses to these challenges and what mechanisms were present and operational in your organization that helped you perform and handle such stresses?

What approaches have you taken to address and incorporate career planning in your responses to those challenges? Have you made any changes to your long term career goals as a result of these experiences?

When it comes to career challenges, have you taken up any creative approaches? In what way have you benefited from these strategies in relation to changes in your career and moving forward?

3. Psychological Reactions

3.1 Fixed Question:

Can you share one of the most emotionally challenging situations you’ve faced in your intership, perhaps involving a difficult case?

3.2 Follow-up Questions:

What strategies did you use to cope with the emotional challenges during that situation?

With expanding experience, physiologically, have you changed the way you experience or address stress? How do you think this affects your professional resilience?

When you feel you are experiencing undue pressure at work, what methods do you tend to employ to relieve yourself of this pressure? Do you change your working conditions or do you resort to other tactics to relieve stress related to work?

After being elevated to the position of a intern, how have you come to balance between schoolwork and life ? How have you found solutions to deal with the conflicts that arise between the two domains?

How has the internship phase of your career affected your personal life, if at all?

4. Behavioral Responses

4.1 Fixed Question:

Can you give detailed examples on how you coped with the professional challenges you confronted or how you overcame them?

4.2 Follow-up Questions:

Please depict a situation in which you had to change your behavioral pattern to get over a professional challenge. What considerations were involved in your reasoning?

In your opinion, how has your approach to managing yourself in stressful situations, changed from the time you started practicing medicine as a intern?

5. External Support

5.1 Fixed Question:

When you encountered difficult situations, what kind of support did you seek from your peer, mentors, or the institutional system?

5.2 Follow-up Questions:

Let us turn to another context and to the specific requirements of the activity you are presenting. Could you describe a specific situation when people from your intership workplace or mentors helped you overcome a very hard situation? Since it was decided, how have their support enabled you to address the situation?

In your opinion, how well did the directions and the feedback you got work for your career struggles? Any specific examples?

How do you view the institutional support that has been extended to you? In what ways has this support been helpful or insufficient for your professional development?

As a new intern, how do you perceive the current healthcare environment? Do you think it provides adequate support for new practitioners like yourself?

Which elements of the healthcare system have had the most significant impact on you as a newly qualified doctor? How have these elements shaped your experience in the early stages of your career?

6. Subjective Needs

6.1 Fixed Question:

Can you outline the future career development needs you expect with regards to resilience that you consider crucial for you as a intern?

6.2 Follow-up Questions:

What in your opinion are resources or competencies that neophytes have to cultivate as a means for survival with the stresses of being foreign practitioners during the intership phase?

What do you think will be the expectations around you assuming your future career specifically, looking into provision of adequate support?

Have you thought about non-traditional methods in dealing with professional setbacks? What other strategies have you used to make progress in your career?

What professional pursuits do you plan to have in the long-range period? How do these plans affect your present-day profession?

At what point have you experienced career-shifting moments? How did these turning points influence your career planning and development?

Probing Questions

Detail-Oriented Probing:

Who were the people involved in the particular situations that you mentioned?

What part did you play in certain clinical or surgical activities?

In which places were these events conducted? Physical or Institutional (settings)?

When did the events take place and what were the time lines in the events?

How were the events, if any, remembered or dealt with after they occurred?

Interpretive Probing:

When you say a task is “difficult” or “ challenging” what do you mean by this? Is it the ‘how’ in the procedure, emotional factors or the environment where it is done?

In instances when phrases like “unfulfilling tasks” are used, what does fulfillment mean to you in this case? Does it imply personal satisfaction, monetary benefits or some other thing?

Clarifying Probing:

When you say “hours worked beyond normal time” do you mean when people have to be brought back to work on days off or holiday or other on other days not scheduled for work? If so, could you please give me more details to be able to tell your story better.

When you say “support from colleagues” do you mean emotional support, professional help, actual help or all of these three things? Why do you need these details?
